# Recognition of Daily Activities in Adults With Wearable Inertial Sensors: Deep Learning Methods Study

**DOI:** 10.2196/57097

**Published:** 2024-08-09

**Authors:** Alberto De Ramón Fernández, Daniel Ruiz Fernández, Miguel García Jaén, Juan M. Cortell-Tormo

**Affiliations:** 1 Department of Computer Technology University of Alicante San Vicente del Raspeig Spain; 2 Department of General Didactics and Specific Didactics University of Alicante San Vicente del Raspeig Spain

**Keywords:** activities of daily living, ADL, ADLs, deep learning, deep learning models, wearable inertial sensors, clinical evaluation, patient’s rehabilitation, rehabilitation, movement, accelerometers, accelerometer, accelerometry, wearable, wearables, sensor, sensors, gyroscopes, gyroscope, monitor, monitoring

## Abstract

**Background:**

Activities of daily living (ADL) are essential for independence and personal well-being, reflecting an individual’s functional status. Impairment in executing these tasks can limit autonomy and negatively affect quality of life. The assessment of physical function during ADL is crucial for the prevention and rehabilitation of movement limitations. Still, its traditional evaluation based on subjective observation has limitations in precision and objectivity.

**Objective:**

The primary objective of this study is to use innovative technology, specifically wearable inertial sensors combined with artificial intelligence techniques, to objectively and accurately evaluate human performance in ADL. It is proposed to overcome the limitations of traditional methods by implementing systems that allow dynamic and noninvasive monitoring of movements during daily activities. The approach seeks to provide an effective tool for the early detection of dysfunctions and the personalization of treatment and rehabilitation plans, thus promoting an improvement in the quality of life of individuals.

**Methods:**

To monitor movements, wearable inertial sensors were developed, which include accelerometers and triaxial gyroscopes. The developed sensors were used to create a proprietary database with 6 movements related to the shoulder and 3 related to the back. We registered 53,165 activity records in the database (consisting of accelerometer and gyroscope measurements), which were reduced to 52,600 after processing to remove null or abnormal values. Finally, 4 deep learning (DL) models were created by combining various processing layers to explore different approaches in ADL recognition.

**Results:**

The results revealed high performance of the 4 proposed models, with levels of accuracy, precision, recall, and *F*_1_-score ranging between 95% and 97% for all classes and an average loss of 0.10. These results indicate the great capacity of the models to accurately identify a variety of activities, with a good balance between precision and recall. Both the convolutional and bidirectional approaches achieved slightly superior results, although the bidirectional model reached convergence in a smaller number of epochs.

**Conclusions:**

The DL models implemented have demonstrated solid performance, indicating an effective ability to identify and classify various daily activities related to the shoulder and lumbar region. These results were achieved with minimal sensorization—being noninvasive and practically imperceptible to the user—which does not affect their daily routine and promotes acceptance and adherence to continuous monitoring, thus improving the reliability of the data collected. This research has the potential to have a significant impact on the clinical evaluation and rehabilitation of patients with movement limitations, by providing an objective and advanced tool to detect key movement patterns and joint dysfunctions.

## Introduction

Activities of daily living (ADL) are the most basic tasks of the person, as they enable them to function with a minimum of autonomy. ADL are crucial for maintaining quality of life and personal well-being, serving as indicators of functional status [1-3]. ADL are an indicator of a person’s functional status and include basic physical tasks such as moving, eating, dressing, maintaining personal hygiene, and grooming, as well as more complex and instrumental activities such as working, shopping, cleaning, exercising, and participating in recreational activities [2-4]. Impaired physical function can limit the execution of these tasks, affecting personal goals and independent living. This condition can affect the individual’s ability to achieve personal goals and maintain an independent quality of life [2,5,6]. Therefore, it is necessary to assess this deterioration during the execution of ADL in different preventive, clinical, or rehabilitation contexts [6-8].

The functional assessment of ADL is complex, so it is advisable to approach it based on the evaluation of fundamental movement patterns on which these ADL are developed [9-11]. The shoulder and lumbar region are key joint complexes in this regard. Specifically, the shoulder joint is essential in many basic ADL, providing the mobility and stability necessary to perform actions in all planes of movement. It is essential to position the hand in space in a way that allows one to reach objects, eat, button a shirt, unbutton a bra, or comb one’s hair [9,12-14]. The movement patterns most used in its assessment are scapula-humeral elevation in the sagittal and frontal plane and rotations at different elevation angles [9,13,14]. Similarly, the lumbar region is a joint complex that has a close relationship with basic movement patterns such as flexion and extension of the trunk in the sagittal plane but also in extremely important actions such as sitting and standing up [10,15-18]. Various ADL derive from this fundamental movement pattern, the most studied being the gestures of sitting and getting up from a chair, bending or crouching, and lifting an object or weight [15-17,19].

The precise evaluation, control, and monitoring of ADL performance are fundamental tasks, although not simple, in the development of effective intervention tools in these clinical and rehabilitation contexts. Traditionally, the assessment of ADL has been based on direct observation and subjective evaluation by therapists, which entails biases, errors, and lack of precision in the results [6,20-22]. In contrast, recent advancements in technology, including wearable health monitoring devices, smart clothing sensors, and mobility assistance devices, enable the objective assessment and quantification of personal performance during ADL [23-27]. This technology includes wearable devices, motion sensors, and 2D or 3D motion capture systems, which allow complex movements and functionality of key joints, such as the shoulder or lumbar region, to be accurately recorded and analyzed during the performance of ADL [4,9,15]. However, limitations such as its high acquisition and implementation cost, its specialized technical knowledge, its lack of transparency and complexity, or its lack of validation and reliability hinder its applicability in the specific clinical or rehabilitation context [4,9,24,25].

A promising solution to overcome the aforementioned limitations is the use of wearable inertial sensors [28-34]. These have been gaining substantial scientific interest due to their potential to provide real-time information on kinematic aspects of human movement through continuous, dynamic, and minimally invasive monitoring. In the clinical and rehabilitation field, this technology has emerged as a simple and low-cost alternative to obtain precise information on accelerations, angular velocities, and trajectories in the different planes of movement during the execution of different basic ADL. This technology offers several advantages. It allows for a more accurate and objective assessment of the functionality of key joint complexes, identifying specific areas of weakness or limitation in movement during ADL and providing quantitative data on the person’s progress over time [28,35,36]. On the other hand, it favors the motivation of patients, by being able to visualize their evolution, thus improving treatment adherence [28,31,37].

However, inertial sensors have some limitations. Despite being light and small, these devices may not be entirely transparent for users, especially due to the high number of sensors that, in many cases, must be used to obtain data that accurately interpret human movement [30,38,39].

Compared with the traditional approach of most studies that only use wearable inertial sensors to monitor kinematic aspects of human movement, the use of artificial intelligence (AI) techniques has been gaining popularity, by helping to improve the process of assessing and supervising different body movements using inertial sensors, in addition to reducing the number of sensors necessary for this [40-42].

In Yen at al [40], a wearable device consisting of a microcontroller and an inertial sensor placed on the participant’s waist is presented. The signals collected by the accelerometer and gyroscope were used to train a 1D convolutional neural network–based feature learning model, enabling the identification of 6 ADL. The results demonstrated high accuracy in both external and study data, validating the effectiveness of the proposed method.

The study by Huynh-The et al [41] introduces an innovative method for recognizing ADL- and sports-related activities using wearable sensors. This method involves converting inertial data into color images, facilitating the learning of highly discriminative features using convolutional neural networks. Experimental results showed recognition accuracy of over 95%, outperforming other deep learning (DL)–based approaches for human activity recognition (HAR).

In Ronald et al [43], a novel DL model inspired by the Inception-ResNet architecture is presented for HAR tasks. The proposed model, trained on data collected from smartphones and inertial sensors capturing accelerometer, gyroscope, magnetometer, GPS, temperature, and heart rate signals, achieved remarkable performance across different data sets, demonstrating its flexibility and adaptability to varying signal types and quantities.

Meanwhile, Poulose et al [44] address the challenges of HAR in health care systems by proposing an approach based on a human image threshing machine using smartphone camera images. The human image threshing system uses mask region–based convolutional neural networks for human detection and a DL model for activity classification, achieving a precision of 98.53% and surpassing conventional sensor-based HAR approaches.

This study is based on the combination of accelerometer and gyroscope signals with AI techniques for the assessment of the shoulder and lumbar spine. AI algorithms can process the data captured by inertial sensors and perform sophisticated analyses to detect patterns, identify alterations in movement, and provide relevant clinical information. This facilitates a more complete and accurate evaluation of the joint movement of the shoulder or lower back, allowing a better understanding of dysfunctions and personalization of treatment and rehabilitation plans. The key contributions made by this study are summarized as follows:

1. Accelerometer and gyroscope signals with AI integration for enhanced ADL assessment: This combination shows great potential for the assessment of shoulder and lumbar region motion in basic ADL performance, providing an objective and advanced perspective in clinical evaluation and rehabilitation. However, validly and reliably demonstrating its use as a control and evaluation tool for ADL performance, in gestures such as eating, combing hair, dressing, sitting, or standing, still appears as an unresolved research challenge. Therefore, in this study, we aim to address the automatic detection and monitoring, using AI techniques, of the patient’s basic ADL related to the shoulder and back.
2. Enhanced activity recognition precision: Our study relies on direct capture of inertial sensor signals, potentially offering a more precise and less image quality–dependent solution.
3. Efficient sensors use: For signal capture, only 2 sensors are used. Furthermore, it is intended to achieve this objective through minimal, noninvasive, and practically transparent sensorization for the user, improving adherence to the monitoring process and facilitating the integration of technology into the individual’s daily life at a low cost.
4. Direct inertial data approach: Our study focuses on the direct use of accelerometer and gyroscope data without requiring additional conversion for model training.
5. Broad scope and versatility: It covers a wide range of activities, showcasing its versatility and adaptability.

We believe that this novel approach will make a significant contribution to this field of research, as it can be used in the prevention, clinical, or rehabilitation contexts of the shoulder and lumbar region.

The remainder of the paper is organized as follows: the *Methods* section addresses how the database was generated, the processing layers used, and the architecture of the 4 developed DL models, as well as the parameters selected for their training and optimization. In the *Results* section, the evaluation outcomes obtained by the 4 DL models are presented, analyzed, and compared. Finally, the *Discussion* section presents a discussion of the principal findings and conclusions regarding our study.

## Methods

### Overview

**Figure 1:**
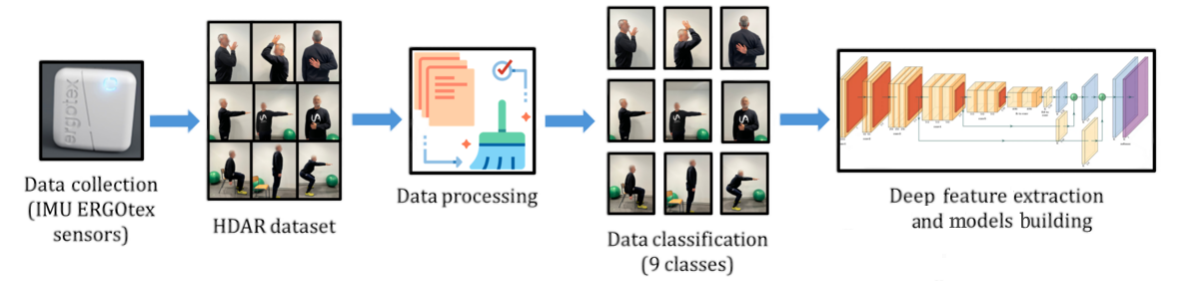
System overview. HDAR: Human Daily Activities Recognition ; IMU: inertial measurement unit.

This research work focuses on the detection and automatic monitoring of ADL using AI models (DL models) and wearable inertial sensors to prevent or diagnose injuries, as well as supervise rehabilitation processes. Figure 1 presents an overview of the methodology proposed. In the following subsections, each step is explained in depth.

### Ethical Considerations

This study has been conducted in strict accordance with the ethical principles outlined in the Declaration of Helsinki. Approval for this research was obtained from the Ethics Committee of the University of Alicante (protocol code UA-2023-11-16).

Prior to commencement, participants provided written informed consent. Respect for participants, including their autonomy, confidentiality, and well-being, has been ensured.

All collected data have undergone a rigorous anonymization process, safeguarding the privacy of the individuals involved in the research. Protective measures were implemented in accordance with institutional guidelines to ensure the security of participant information throughout the study.

Participants involved in human subjects research were not provided with any form of compensation. This decision was made to uphold transparency and fairness in the research process and to minimize potential biases associated with compensation.

### Data Collection

**Figure 2:**
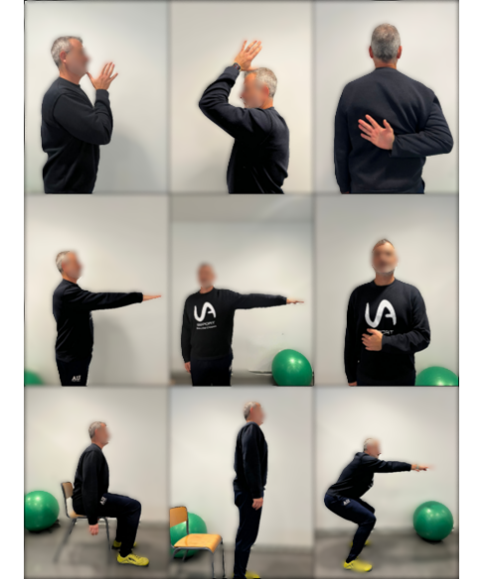
Graphic description of activities of daily living movements. In the top row (from left to right): eating, combing hair, and fastening the bra. In the middle row (from left to right): opening the door, reaching for an object, and buttoning up. Bottom row (left to right): sitting, standing up, and half squat.

A total of 9 ADLs were included in the study, 6 of them related to the shoulder (eating [E], combing hair [CH], fastening the bra [FB], opening the door [OD], reaching for an object [RO], and buttoning up [BU]) and 3 related to the back (sitting [S], standing up [SU], and half squat [HS]). Figure 2 graphically shows these movements.

**Figure 3:**
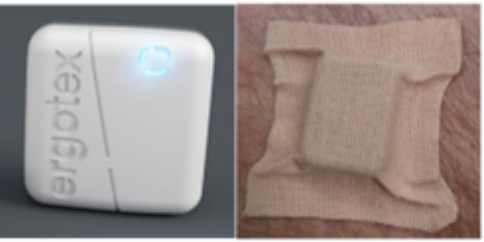
ERGOtex inertial measurement unit sensors were developed for movement identification.

To monitor movements, we used 2 self-developed inertial measurement unit ERGOtex model sensors [45,46]. This inertial measurement unit ERGOtex sensor comprises 3 triaxial accelerometers (±2 g, controlled noise at 100 µg/√Hz), triaxial gyroscopes (±1000 deg/s, sensitivity error within ±1%, and low noise level, at ±4 mdeg/s/√Hz), and magnetometers, encapsulated in a device (weight=8 g, dimensions=23×21×10 mm). The ICM-20602 MEMS MotionTracking (TDK Corp) device was selected for its high-performance specifications, critical for the reliability of the device. The incorporation of a 1K-byte FIFO buffer reduces serial bus congestion, enhancing measurement consistency and optimizing device power use. It operates at a sampling rate of 20 Hz, has an autonomy of 8 hours, and can be attached to the skin using double-sided tape or secured elsewhere using an elastic strap. These enhancements guarantee reliable response times and sensitivity levels, crucial for maintaining data accuracy (Figure 3).

The inertial sensors were attached to the skin over the sacrum (S1) and the distal part of the upper extremity (close to the wrist). Primarily designed for monitoring spine posture and arm, this device records acceleration data across all 3 axes. Internal integration of the acceleration signal occurs within the device, transmitting data instantly via Bluetooth (frequency=2.4 GHz) to a smartphone or tablet equipped with the preinstalled app. This application enables immediate data visualization and facilitates export to a spreadsheet in comma-separated text format (CSV).

The database generated initially had 53,165 records of all activities. The records were grouped into batches of time series (of different lengths) that represented the different movements. Each record was made up of 12 attributes or numerical variables, corresponding to the value obtained by the accelerometer and gyroscope of each sensor during the execution of the movement according to its 3 axes (Acx, Acy, Acz, Gyx, Gyy, Gyz). After the processing stage, where null, missing, and abnormal values were eliminated, the database was reduced to 52,600 records (RO: n=6423, FB: n=6956, E: n=6216, OD: n=6472, SU: n=3678, CH: n=6010, BU: n=5915, HS: n=6630, and S: n=4300).

### DL Models

#### Processing Layers

To create the DL models, different processing layers that perform the transformation, regularization, feature extraction, regularization, and dependency capture operations were combined. The basics of each of them are presented below.

#### 1D Convolution Layer for Feature Extraction

A 1D convolutional layer is specifically designed to process data that follows a 1D structure, such as time series or text sequences. In the case of a 1D time series, the 1D convolution operation follows a similar process as a standard convolutional layer but is performed along 1 dimension instead of 2 [47]. The convolution operation is the key component of this type of layer. During the 1D convolution operation, a filter (kernel) of defined size slides along the time series, multiplying the filter values by the corresponding values in the time series and summing them to produce a single value at the output. This process is repeated for each filter position throughout the time series, thus generating a feature map that highlights relevant patterns in the data sequences. The 1D convolutional layer is essential for the automatic identification of patterns in time series, allowing efficient extraction of important features during the training process. By reducing the number of parameters and avoiding overfitting, 1D convolution helps capture the temporal structure of data and improve model performance in time series prediction or classification tasks [48]. Given an input 1D time series *X* and a set of filters *F*, the convolution operation is performed as follows (equation 1):

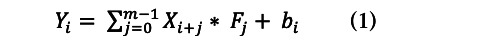



where *Y_i_* is the output value at the feature map position *i*, *X_i+j_* is the time series value at position *i+j*, * denotes the convolution operation, and *b_i_* is the bias associated with the output *F_j_*, and *m* is the filter size.

#### Long Short-Term Memory Layer for Modeling Temporal Dependencies

Long short-term memory (LSTM) layers are a type of recurrent layer designed to overcome the limitations of traditional recurrent neural networks in capturing long-term dependencies in temporal sequences [49]. Its design is based on the idea of using internal memory structures controlled by gates to manage information over time and make decisions about what information to retain and discard. In an LSTM, 3 main gates are introduced: the forget gate, which decides what information should be discarded from the previous memory; the input gate, which decides what new information should be stored in memory; and the output gate, which determines what memory information should be used to generate the output of the layer. These gates are controlled by activation functions and adjustable weights during training.

An overview of the fundamental equations of an LSTM cell is presented below, which describe how an LSTM cell manages information and gates to process and retain relevant information over time in a temporal sequence, given one input at a time step *t*, denoted as *x_t_*, and the outputs of the previous time step [*h_t_*_–1_] (LSTM cell output) and *C_t_*_–1_ (LSTM cell state).

Forget gate (*f_t_*): decides what information should be discarded or forgotten from the cell state (equation 2)

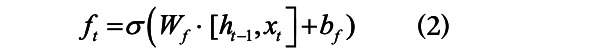



Input gate (*i_t_*): decide what new information to store in the cell state (equations 3 and 4)

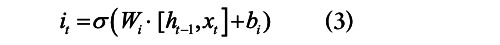

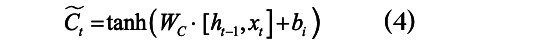





The forgotten information and new information are then combined to update the state of the cell (equation 5).

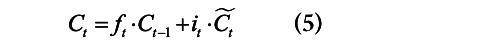



Output gate (*o_t_*): finally, the final activation at the current position (*h_t_*) is calculated with the output gate (*o_t_*), which regulates the amount of information to be output (equation 6)

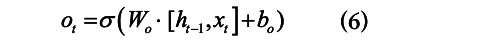

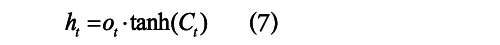





Where σ is the sigmoid function; *tanh* is the hyperbolic tangent function; *W_i_*,*W_C_* and *W_o_* are weight matrices that are learned during training; and *b_f_, b_i_, b_c_,* and *b_o_* are biases. [*h_t–_*_1_,*x_t_*] denotes the concatenation of *h_t–_*_1_ and *x_t_* before applying the linear operation.

#### Dropout Regularization Layer

The Dropout layer is a regularization strategy that prevents overfitting by introducing variability into the network during training [50]. This technique randomly turns off a percentage of units in each iteration, temporarily removing them and forcing the network to learn more robust representations. Based on the assembly concept, it simulates the presence or absence of units, improving effectiveness and reducing dependence on specific units. In addition to its impact on generalization, the Dropout layer acts as an effective regularization mechanism, improving modeling efficiency and performance by preventing overoptimization and facilitating generalization to unseen data [51,52].

#### Flatten and Fully Connected (Dense) Transformation Layers

The Flatten layer aims to transform 2D or 3D data into a 1D format, allowing for a more manageable representation and facilitating the transition from convolutional layers to dense layers [53]. Given a 3D input matrix where *m*, *n*, and *p* are the spatial dimensions, the Flatten layer converts this matrix into a 1D vector *X’* of size *m* * n * p.

The fully connected (FC) or Dense layer connects all neurons in 1 layer to all neurons in the next layer [48]. It performs linear transformations on the data followed by nonlinear activation functions, allowing complex representations to be learned. If *X* is the input of the Dense layer, *W* is the weight matrix, and *b* is the bias vector, the output *Y* is calculated as (equation 8):

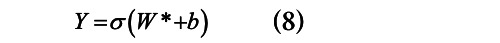



where σ is the activation function.

#### 1D MaxPooling Layer for Feature Reduction

The 1D MaxPooling layer is a technique used in neural networks to reduce the spatial dimensionality of data by retaining only the maximum values in specific regions [54,55]. In the context of 1D time series, 1D MaxPooling is used to summarize the most relevant information and reduce the computational cost by decreasing the number of parameters in the network. Given a 1D input data set *X* with elements and a pooling window of size *p*, the output *Y* is calculated by taking the maximum value in each window. Mathematically, this can be expressed as (equation 9):

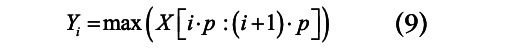



where *i* is the index of the pooling window. This process is repeated until the entire length of the entry is covered.

#### Proposed Architecture

#### Overview

The processing layers described above were combined to create 4 DL models of different complexity. Each model was designed to explore and exploit different approaches in data processing for the ADL recognition task. The architectures and distinctive features of each of these models are detailed below.

#### Convolutional Approach

**Figure 4:**
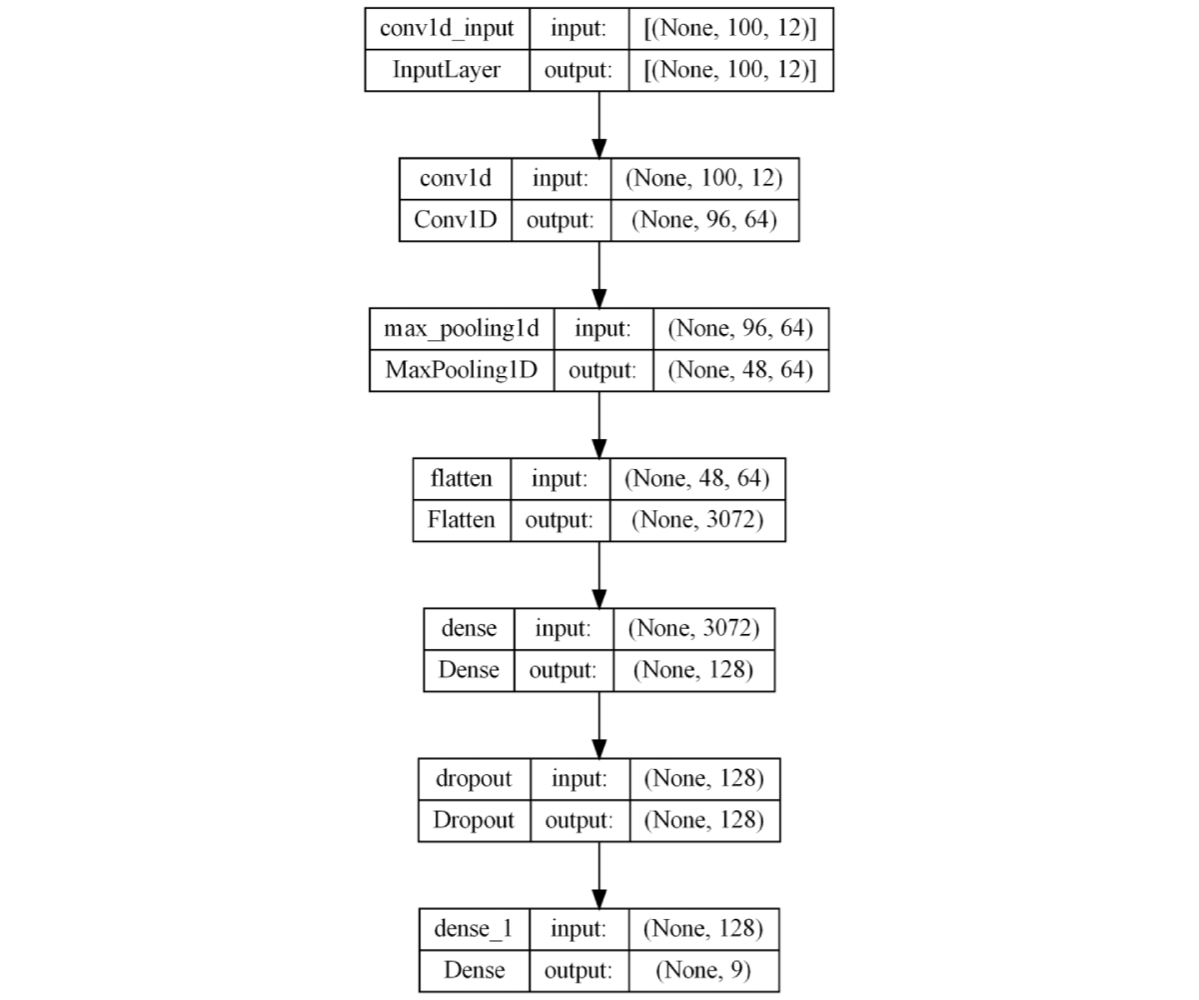
Model architecture based on a convolutional approach.

The first proposed architecture uses a convolutional approach. It is composed of 3 main layers: a 1D convolutional layer, a pooling layer, and an FC layer (Figure 4). The convolutional layer, with 64 filters and a kernel size of 5, performs local feature extraction. Next, the pooling layer with pool size 2 is applied to reduce the dimensionality and preserve the most relevant features. Subsequently, a Flatten layer is used to convert the output into a 1D vector before connecting it to an FC layer with 128 neurons and a rectified lineal unit (ReLU) activation function. ReLU is a nonlinear activation function commonly used in neural networks to introduce nonlinearities and aid in model convergence [56]. Finally, a Dropout layer with a rate of 40% is incorporated to prevent overfitting. The output layer uses the Softmax function and is designed for multiclass classification. The output layer uses the Softmax function, which is commonly used in multiclass classification tasks to compute the probabilities of each class outcome and facilitate decision-making based on the highest probability class [57].

#### Deep LSTM Approach

**Figure 5:**
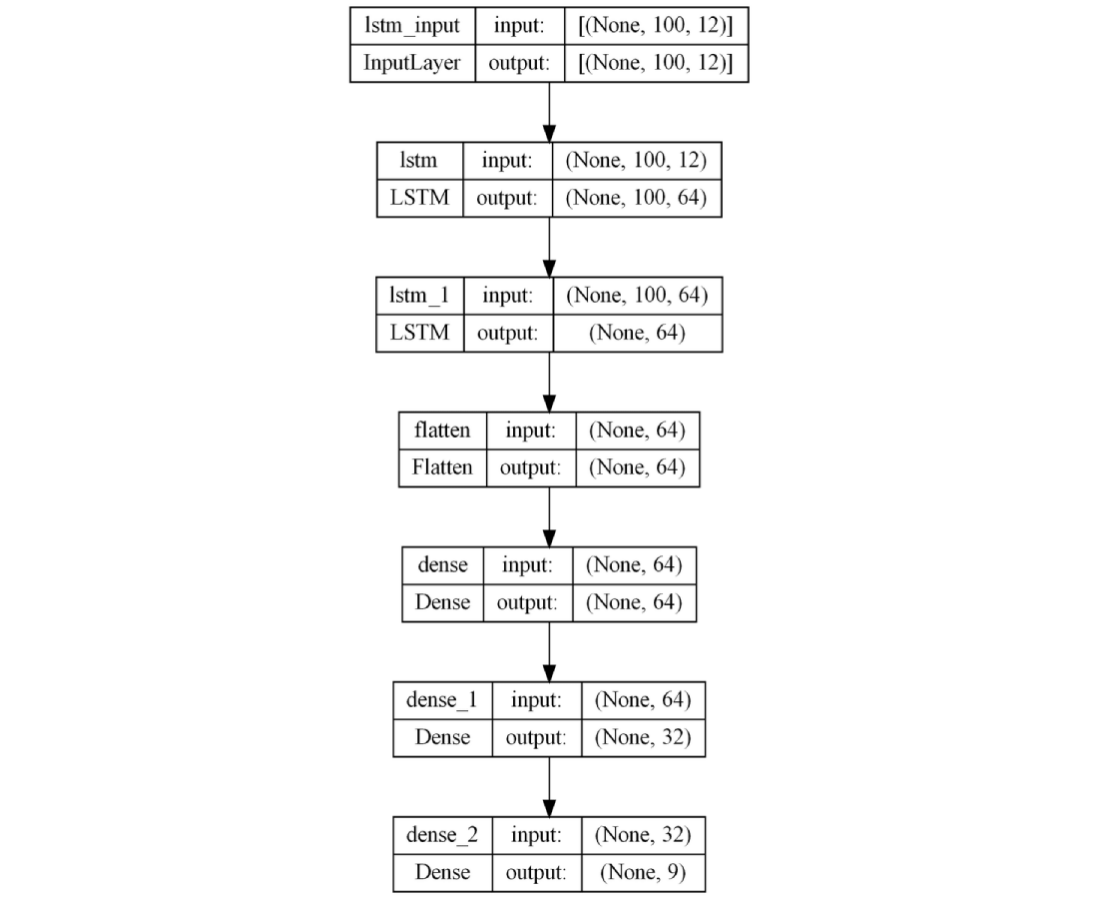
Model architecture based on a deep LSTM approach. LSTM: long short-term memory.

The second architecture is based on a deep LSTM networking approach. It includes 2 LSTM layers, both with 64 units, followed by a Flatten layer. Then, 2 FC layers, with 64 and 32 neurons, respectively, and ReLU activation function are incorporated. The output layer uses the Softmax function for multiclass classification (Figure 5). This architecture deepens into the LSTM network with multiple layers, allowing more complex temporal patterns to be learned. The complexity increases compared with the convolutional model due to the deepening of the LSTM layers and the increase in FC connections. This approach seeks to capture more elaborate temporal dependencies in time series data.

#### Hybrid Approach: 1D Convolutional + LSTM

**Figure 6:**
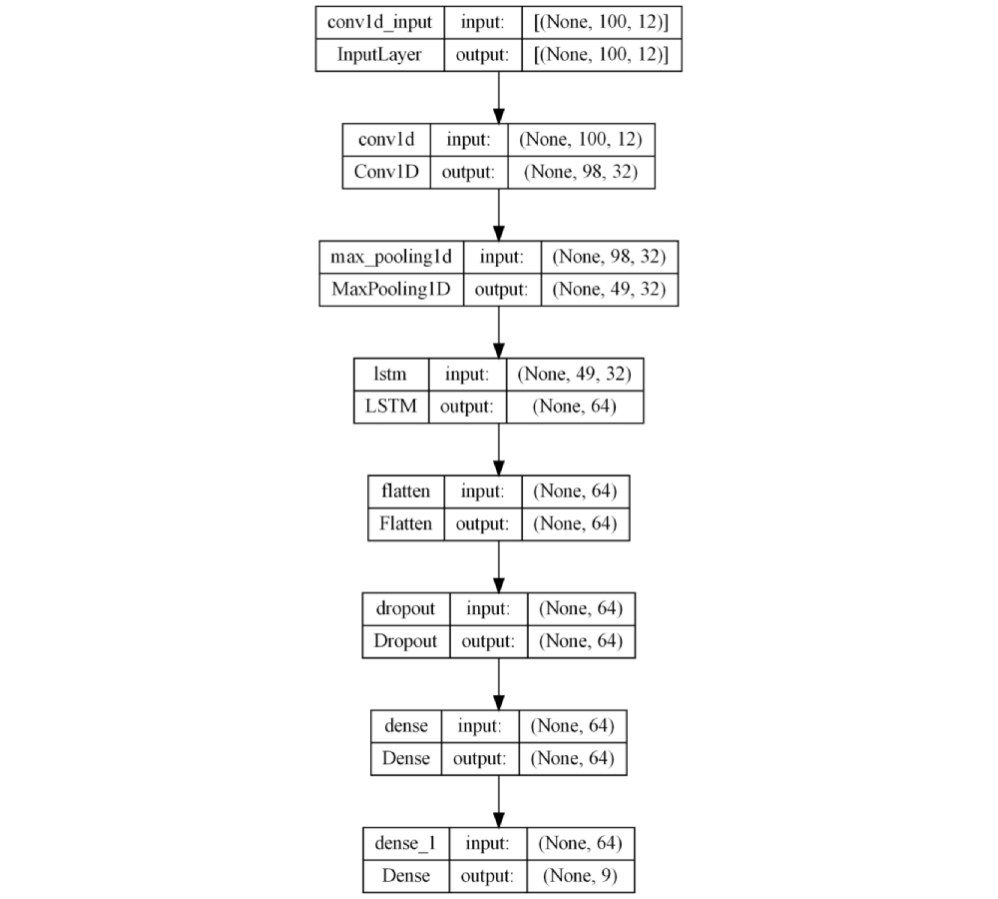
Model architecture based on a hybrid approach (convolutional + LSTM). LSTM: long short-term memory.

The third architecture adopts a hybrid approach combining convolutional layers and LSTM networks (Figure 6). It starts with a 1D convolutional layer with 32 filters and kernel size 3, followed by an LSTM layer with 64 units. Subsequently, a pooling layer and a Flatten layer are applied. A Dropout layer (30%) is introduced to prevent overfitting before connecting to an FC layer with 64 neurons and ReLU activation. The output layer uses Softmax for multiclass classification. This architecture seeks to take advantage of the ability of convolutional layers to extract local features and the ability of LSTMs to model long-term temporal dependencies, offering a combination of both capabilities. Its complexity lies in the integration of 2 different approaches to improve the representation and understanding of time series data.

#### Bidirectional LSTM Approach

**Figure 7:**
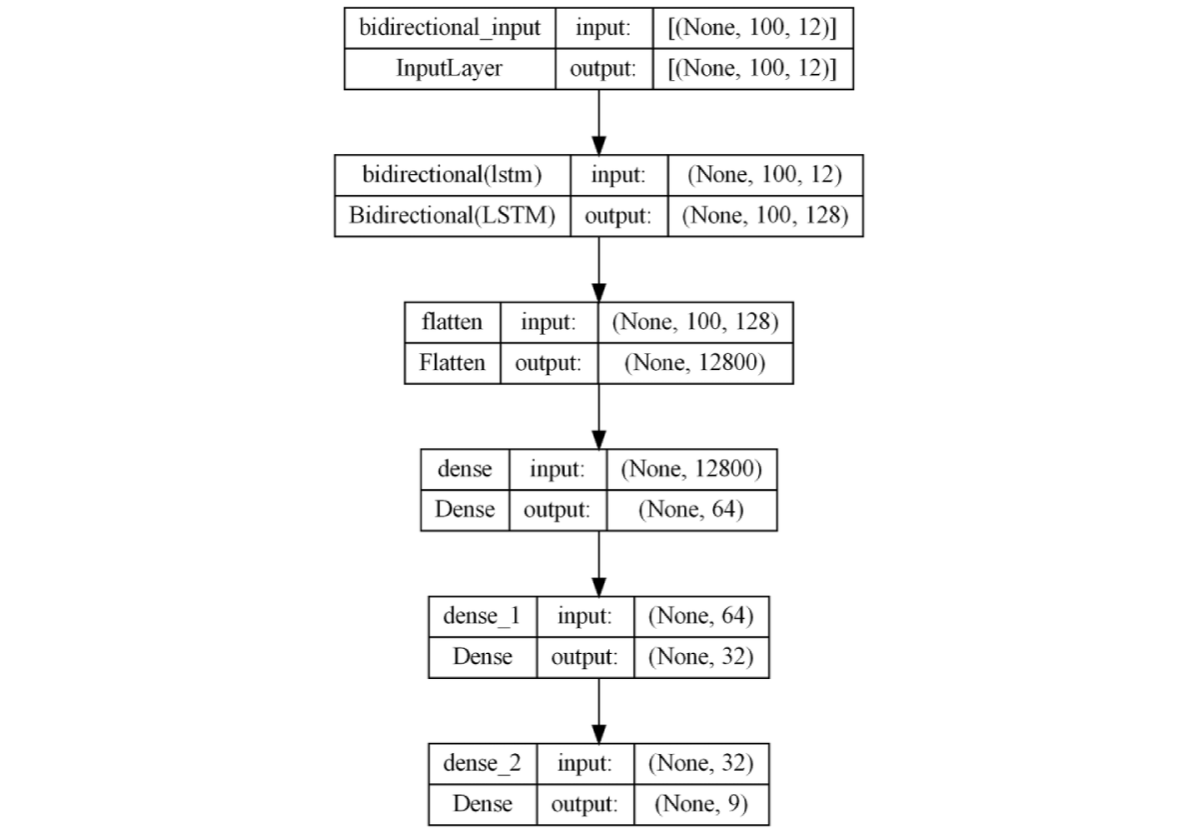
Model architecture based on a bidirectional LSTM approach. LSTM: long short-term memory.

The fourth architecture adopts a bidirectional approach using LSTM layers (Figure 7). It starts with a bidirectional LSTM layer with 64 units to capture temporal patterns in both directions. Then, a Flatten layer is applied before connecting with 2 FC layers of 64 and 32 neurons, respectively, with ReLU activation function. The output layer uses Softmax for multiclass classification. This architecture represents a more sophisticated and complex model by taking advantage of the ability of bidirectional LSTMs to capture both forward and backward temporal dependencies.

#### Selection of Parameters for Training and Optimization of Models

**Figure 8:**
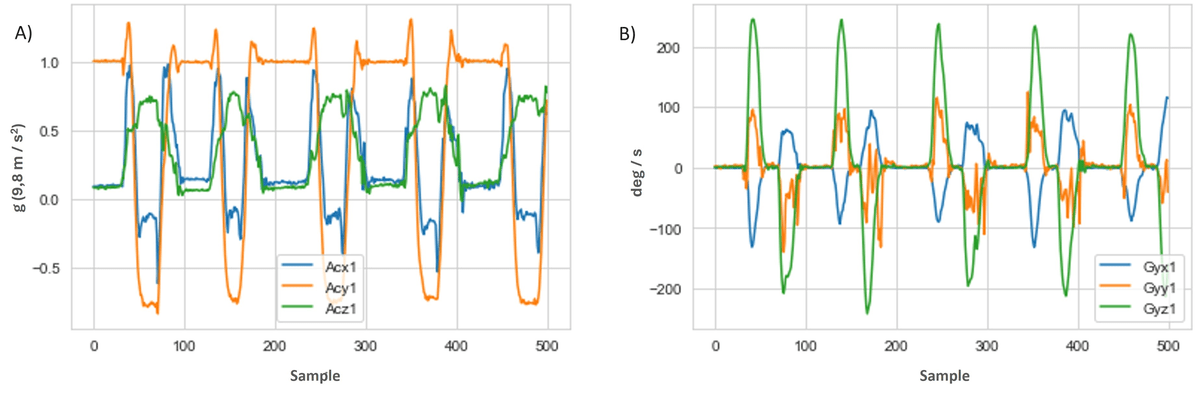
Time series of eating activity: (A) accelerometer and (B) gyroscope.

For a better understanding of the data and selection of the hyperparameters of the AI model, the accelerometry and gyroscope values of each movement were analyzed separately (Figure 8). Based on this, the temporal sequences were divided into windows of 100 records with a 10-record overlap between adjacent windows.

Each model was trained over 150 epochs, representing a complete iteration through the training data. The model weights were updated every 1024 records (batch size), and training was stopped if the validation accuracy did not improve for 15 consecutive epochs (early stopping) to prevent overfitting.

As the optimization algorithm during training, Adam was used. Its primary goal is to adjust the network’s weights and biases so that the model’s loss function is minimized. Adam enhances the standard gradient descent technique by adjusting the learning rate for each parameter individually, potentially leading to faster convergence and better model performance.

Additionally, an L2 regularization term with a strength of 0.0015 was also applied to mitigate overfitting. This term controls the excessive growth of weights during training by adding a penalty term to the model’s loss function. The regularization strength determines how much large weights are penalized. By penalizing large weights, L2 regularization helps smooth out the model’s decisions and prevents it from fitting too closely to the training data.

Categorical cross-entropy was used as the loss function. This function measures the discrepancy between the probability distributions predicted by the model and the actual distributions. The primary evaluation metric was accuracy, indicating the proportion of the model’s predictions in the test set that were correct.

## Results

### Evaluation Metrics

The experiment was performed on a personal computer with Microsoft Windows 10, an Intel(R) Core(TM) i5-4210U CPU @ 1.70GHz-2.40 GHz, 6 GB RAM, and no GPU. All software was implemented using the Python programming language and the TensorFlow library in the Spyder development environment. After preparing the data, the DL models were trained using 70% of the data, and the remaining 30% was used to evaluate their performance. For this, popular evaluation metrics were used in classification problems, including precision, recall, *F*_1_-score, and accuracy.

Accuracy (equation 10) refers to the proportion of correct predictions, true positives (TPs) and true negatives (TNs) in relation to the total predictions made by the model, which include false positives (FPs) and false negatives (FNs).

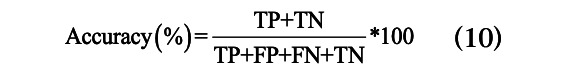



Precision (equation 11) represents the proportion of positive predictions that were correct. It is calculated as the number of TPs divided by the sum of TPs and FPs.

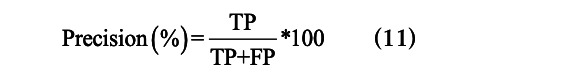



Recall (equation 12) refers to the proportion of TP cases that were correctly identified by the model, calculated as the number of TPs divided by the sum of TPs and FNs.

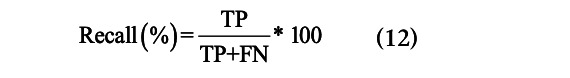



*F*_1_-score (equation 13) is a measure that combines precision and recall. It is calculated as the harmonic mean between precision and recall and provides a more balanced assessment of model performance, particularly useful when there is an imbalance in the class distribution in the data.

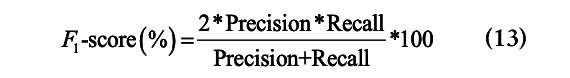



### Evaluation Outcomes

The obtained results show the high performance of the 4 proposed models, with accuracy, precision, recall, and *F*_1_-score ranging between 95% and 97% for all cases (Table 1), while the loss function indicates an error rate of approximately 0.10 for the models. The high accuracy, precision, and recall suggest an ability to accurately identify multiple classes of activities, while the high *F*_1_-score indicates a good balance between precision and recall. These results suggest that the models have effectively learned the relationships in the training, enabling them to identify patterns and generalize effectively to data they have not encountered during training, demonstrating strong and reliable predictive capabilities.

**Table 1:** Evaluation metrics of the designed deep learning models.

Models	Accuracy (%)	Precision (%)	Recall (%)	*F*_1_-score (%)
CNN^a^	97.11	97.19	97.14	97.14
Deep LSTM^d^	95.52	95.64	95.58	95.54
CNN+LSTM	96.19	96.19	96.14	96.14
Bidirectional LSTM	97.56	97.51	97.52	97.51

^a^CNN: convolutional neural network.

^b^LSTM: long short-term memory.

**Figure 9:**
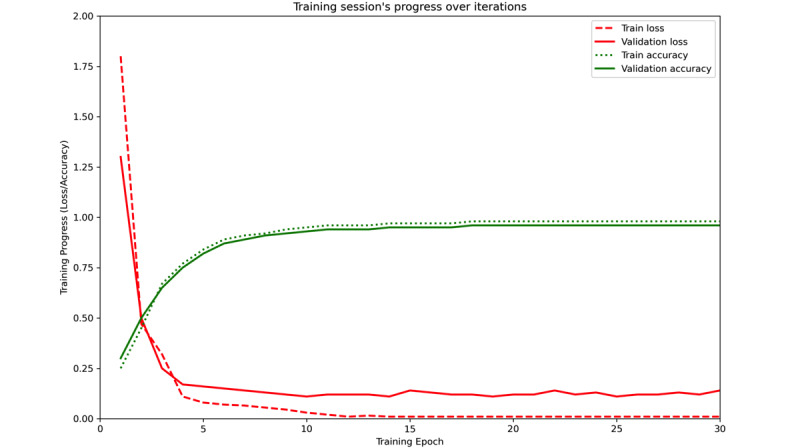
Training sessions progress over iterations.

When comparing different modeling approaches, it is evident that both the convolutional and bidirectional methods yield similar results across all evaluated metrics. This suggests that, despite the bidirectional approach’s inherent complexity in processing sequences in both directions, it does not offer a significant improvement over the simpler convolutional method. The convolutional model may have struck an optimal balance between learnability and generalization, enabling it to match or even surpass more complex models in terms of accuracy. However, it is worth noting that the bidirectional model achieved convergence in a smaller number of epochs (n=30; Figure 9), which is particularly valuable when rapid training and model responsiveness are required.

It is also noteworthy that more complex models, such as deep LSTM and the hybrid approach, exhibit slightly inferior results compared with the convolutional approach. This observation may stem from several factors. First, the generalization ability of these models may be compromised due to the inherent complexity of their architectures and sensitivity to weight initialization. Additionally, the nature of the data and the suitability of different modeling approaches to capture the relevant characteristics of the time series should be considered. The activities represented in the data may benefit more from a simpler, more straightforward approach, such as convolutional, rather than more complex methods that may be prone to capturing irrelevant features or noise in the data.

**Figure 10:**
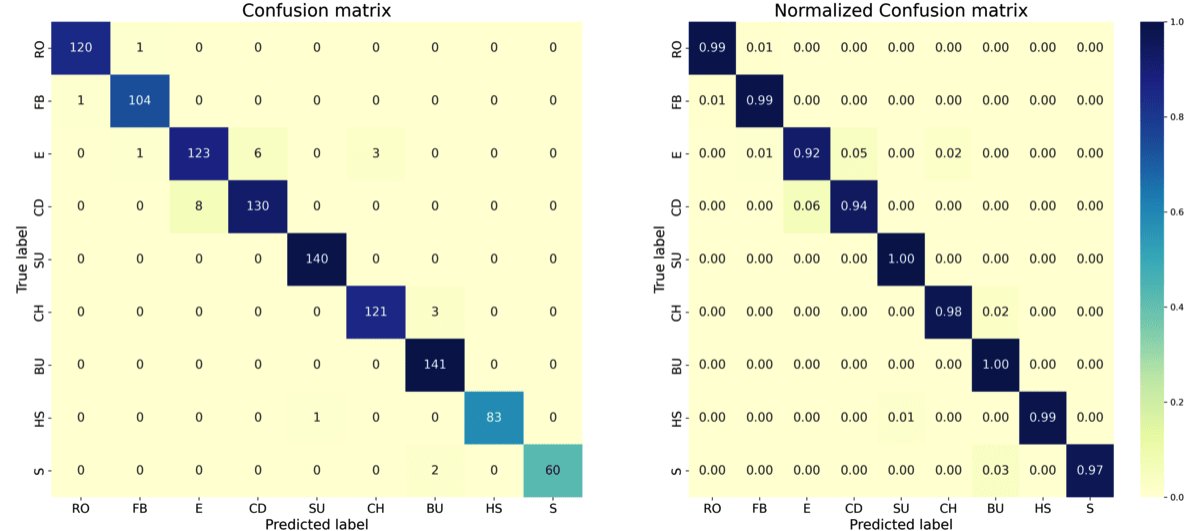
Confusion matrix of the winning model based on the bidirectional approach: (left) standard and (right) normalized. BU: buttoning up; CH: combing hair; E: eating; FB: fastening the bra; HS: half squat; OD: opening the door; RO: reaching for an object; S: sitting SU: standing up.

At the activity or class level, the confusion matrix provides a detailed breakdown of the model predictions for each class compared with the real class. Referring to the confusion matrix of the model with the best performance (Figure 10), it is observed that the majority of the predictions align with the main diagonal of the matrix, indicating that, for the most part, the classes are classified correctly. However, the activity of eating exhibits the most erroneous predictions, primarily being confused with the activities of opening a door and combing one’s hair. This confusion may arise due to overlapping movements and shared characteristics, such as acceleration and rotation patterns, making it challenging for the model to distinguish between them. Moreover, variations in the sequence of movements and the context in which these activities are performed may lead to different interpretations by the model. Variability in the execution of activities and differences in movements between individuals can also contribute to confusion among these classes.

## Discussion

### Background

ADL are fundamental tasks that enable individuals to function with a minimum of autonomy and maintain their quality of life. Precise evaluation of ADL, especially in clinical and rehabilitation contexts, is crucial for understanding individuals’ functional status and designing effective interventions. Traditionally, the assessment of ADL has relied on direct observation and subjective evaluation by therapists, which can lead to biases and errors. Innovative technology, including wearable inertial sensors and AI, offers new opportunities for objective and quantitative evaluation of ADL performance.

### Principal Findings

This study presents an innovative initiative by combining wearable inertial sensors with AI techniques to evaluate human movement in ADL. The implemented AI models have demonstrated solid performance, exhibiting high accuracy, precision, recall, and *F*_1_-score (ranging between 95% and 97%), indicating an effective ability to identify and classify a variety of daily activities related to the shoulder and lumbar region. Furthermore, these results have been achieved through minimal sensorization, which is noninvasive and practically imperceptible to the user, thus minimizing interference with their daily life. This feature is crucial as it promotes user acceptance and adherence to continuous monitoring, contributing to the reliability of the collected data.

### Comparison to Prior Work

This study presents significant improvements in the identification and monitoring of activities of ADL compared with other existing methods. Unlike most previous approaches that primarily focus on activities involving the lumbar region (sitting, lying down, standing up, etc), our proposal allows for the precise identification of complex movements involving both the lumbar region and the shoulder. This is achieved using only 2 low-cost inertial sensors, contrasting with other solutions that require a higher degree of sensorization or bulkier devices. This minimally invasive monitoring enables individuals to perform daily activities naturally, promoting a more authentic representation of movement.

The information provided by the sensors is used by DL algorithms for movement identification, without requiring additional processing. This enables immediate analysis of movement patterns during the performance of everyday activities, avoiding the delay associated with data processing needed in image-based motion capture systems, which tend to be more expensive and complex to set up and maintain.

Furthermore, the use of inertial sensors offers versatility and adaptability, making them suitable for monitoring a wide range of ADL in different environments and contexts. They provide valuable information on movement patterns and functional abilities that may not be effectively captured or may be more difficult to capture by traditional 2D and 3D motion capture systems, which are more limited by factors such as image quality, potential obstructions in the line of sight between the camera and the person, or the need to use a greater number of cameras or sensors to capture all movement details.

### Limitations and Strengths of This Study

This study demonstrates notable strengths in its methodology and approach. It uses the integration of inertial sensors and AI to improve the assessment of shoulder and lumbar motion during basic ADL performance, providing an objective and advanced perspective for clinical evaluation and rehabilitation. Although challenges persist in validating its use across various ADL gestures, such as eating or dressing, our focus on automatic detection and monitoring using AI techniques addresses this gap. Furthermore, by directly capturing inertial sensor signals and using only 2 sensors, our approach ensures enhanced activity recognition precision and efficiency. This strategy facilitates seamless integration into individuals’ daily lives at a low cost, promoting improved adherence to monitoring. Additionally, our study’s direct use of accelerometer and gyroscope data without conversion for model training emphasizes its versatility and broad scope, highlighting its adaptability across a wide range of activities.

However, it is essential to acknowledge potential limitations to encourage further research and refinement. One limitation lies in the scope of activities monitored, which primarily focuses on specific muscle groups. Future research should aim to expand the scope of using AI and wearable inertial sensors beyond the assessment of shoulder and lumbar motion, broadening the range of monitored ADL. Given this limitation, it would be interesting to conduct in the future more extensive studies that encompass a broader range of ADL and other more distal body segments. For instance, investigations could explore the application of these technologies in assessing motion patterns related to limb motion (ie, elbow and wrist, or knee and ankle movements), offering valuable insights into biomechanical segmentary dynamics and enhancing our understanding of musculoskeletal movement patterns through AI approaches. Despite this limitation, the study sets a solid foundation for future endeavors in this field, showcasing its potential for advancement and application in clinical and rehabilitative settings.

### Conclusions

This research has the potential to significantly impact the clinical evaluation and rehabilitation of patients with movement limitations, offering an objective and advanced tool to detect key movement patterns and joint dysfunctions. Such information can assist professionals in tailoring treatment plans to be more precise and personalized, addressing specific areas of weakness, and designing interventions to improve the patient’s functionality and quality of life.

## Abbreviations

ADL: activities of daily living
AI: artificial intelligence
BU: buttoning up
CH: combing hair
DL: deep learning
E: eating
FB: fastening the bra
FC: fully connected
FN: false negative
FP: false positive
HAR: human activity recognition
HS: half squat
OD: opening the door
LSTM: long short-term memory
RO: reaching for an object
ReLU: rectified lineal unit
S: sitting
SU: standing up
TN: true negative
TP: true positive

## Data Availability

The datasets generated during this study are not publicly available due to ethical restrictions but are available from the corresponding author on reasonable request.

## References

[1] Edemekong PF, Bomgaars D, Sukumaran S, Levy SB (2019). Activities of Daily Living.

[2] Merrilees J (2014). Activities of daily living. Encyclopedia of the Neurological Sciences.

[3] Katz S (1983). Assessing self-maintenance: activities of daily living, mobility, and instrumental activities of daily living. J Am Geriatr Soc.

[4] Kang H, Lee C, Kang SJ (2023). A smart device for non-invasive ADL estimation through multi-environmental sensor fusion. Sci Rep.

[5] Chan CS, Slaughter SE, Jones CA, Wagg AS (2015). Greater independence in activities of daily living is associated with higher health-related quality of life scores in nursing home residents with dementia. Healthcare (Basel).

[6] Giebel CM, Sutcliffe C, Challis D (2015). Activities of daily living and quality of life across different stages of dementia: a UK study. Aging Ment Health.

[7] Herero VG, Extremera N (2010). Daily life activities as mediators of the relationship between personality variables and subjective well-being among older adults. Pers Individ Differ.

[8] Osborne M, Rizzo J (2007). Chapter 106 - neurorehabilitation. Neurol Clin Neurosci.

[9] Klemt C, Prinold JA, Morgans S, Smith SHL, Nolte D, Reilly P, Bull AMJ (2018). Analysis of shoulder compressive and shear forces during functional activities of daily life. Clin Biomech (Bristol, Avon).

[10] Vaisy M, Gizzi L, Petzke F, Consmüller T, Pfingsten M, Falla D (2015). Measurement of lumbar spine functional movement in low back pain. Clin J Pain.

[11] Kaljić E, Pašalić A, Katana B, Mačak Hadžiomerović A, Bojičić S, Jaganjac A, Salkić N (2022). Influence of motion therapy on daily life activities of people with lumbar pain syndrome. J Health Sci.

[12] Poppen N K, Walker P S (1976). Normal and abnormal motion of the shoulder. J Bone Joint Surg Am.

[13] Magda A, Cáceres L (2019). Doctoral Thesis. University of Valencia.

[14] Michiels I, Grevenstein J (1995). Kinematics of shoulder abduction in the scapular plane. on the influence of abduction velocity and external load. Clin Biomech (Bristol, Avon).

[15] Sánchez-Zuriaga D, López-Pascual J, Garrido-Jaén D, de Moya MFP, Prat-Pastor J (2011). Reliability and validity of a new objective tool for low back pain functional assessment. Spine (Phila Pa 1976).

[16] Artacho PCA, Andrea C (2018). Biomechanical assessment of the spine based on functional analysis of various activities of daily living. University of Valencia.

[17] Fuster Ortí MA (2021). Effects of a manual spinal traction technique on the lumbo-pelvic movement pattern and activation of the erector spinae during trunk flexion-extension in patients with low back pain. University of Valencia.

[18] Lehman GJ (2004). Biomechanical assessments of lumbar spinal function. how low back pain sufferers differ from normals. implications for outcome measures research. part i: kinematic assessments of lumbar function. J Manipulative Physiol Ther.

[19] Arguisuelas MD, Lisón JF, Doménech-Fernández J, Martínez-Hurtado I, Salvador Coloma P, Sánchez-Zuriaga D (2019). Effects of myofascial release in erector spinae myoelectric activity and lumbar spine kinematics in non-specific chronic low back pain: randomized controlled trial. Clin Biomech (Bristol, Avon).

[20] Katz S, Ford AB, Moskowitz RB, Jackson BA, Jaffe MW (1963). Studies of illness in the aged: the index of ADL: a standardized measure of biological and psychosocial function. JAMA J Am Med Assoc.

[21] Morris JN, Fries BE, Morris SA (1999). Scaling ADLs within the MDS. J Gerontol A Biol Sci Med Sci.

[22] Graf C, Hartford Institute for Geriatric Nursing (2008). The Lawton instrumental activities of daily living (IADL) scale. Medsurg Nurs.

[23] Sánchez-Zuriaga D, Artacho-Pérez C, Biviá-Roig G (2016). Lumbopelvic flexibility modulates neuromuscular responses during trunk flexion-extension. J Electromyogr Kinesiol.

[24] Pashmdarfard M, Azad A (2020). Assessment tools to evaluate activities of daily living (ADL) and instrumental activities of daily living (IADL) in older adults: a systematic review. Med J Islam Repub Iran.

[25] Jekel K, Damian M, Storf H, Hausner L, Frölich L (2016). Development of a proxy-free objective assessment tool of instrumental activities of daily living in mild cognitive impairment using smart home technologies. J Alzheimers Dis.

[26] Amaral Gomes ES, Ramsey KA, Rojer AGM, Reijnierse EM, Maier AB (2021). The association of objectively measured physical activity and sedentary behavior with (instrumental) activities of daily living in community-dwelling older adults: a systematic review. Clin Interv Aging.

[27] Goverover Y, Kalmar J, Gaudino-Goering E, Shawaryn M, Moore NB, Halper J, DeLuca J (2005). The relation between subjective and objective measures of everyday life activities in persons with multiple sclerosis. Arch Phys Med Rehabil.

[28] Bonato P (2005). Advances in wearable technology and applications in physical medicine and rehabilitation. J Neuroeng Rehabil.

[29] Kim J, Campbell AS, de Ávila BEF, Wang J (2019). Wearable biosensors for healthcare monitoring. Nat Biotechnol.

[30] Rodgers MM, Alon G, Pai VM, Conroy RS (2019). Wearable technologies for active living and rehabilitation: current research challenges and future opportunities. J Rehabil Assist Technol Eng.

[31] Lang CE, Barth J, Holleran CL, Konrad JD, Bland MD (2020). Implementation of wearable sensing technology for movement: pushing forward into the routine physical rehabilitation care field. Sensors (Basel).

[32] Porciuncula F, Roto AV, Kumar D, Davis I, Roy S, Walsh CJ, Awad LN (2018). Wearable movement sensors for rehabilitation: a focused review of technological and clinical advances. PM R.

[33] Jalloul N (2018). Wearable sensors for the monitoring of movement disorders. Biomed J.

[34] Wu W, Dasgupta S, Ramirez EE, Peterson C, Norman GJ (2012). Classification accuracies of physical activities using smartphone motion sensors. J Med Internet Res.

[35] Rast FM, Labruyère R (2020). Systematic review on the application of wearable inertial sensors to quantify everyday life motor activity in people with mobility impairments. J Neuroeng Rehabil.

[36] Kristoffersson A, Lindén M (2022). A systematic review of wearable sensors for monitoring physical activity. Sensors (Basel).

[37] Camomilla V, Bergamini E, Fantozzi S, Vannozzi G (2018). Trends supporting the in-field use of wearable inertial sensors for sport performance evaluation: a systematic review. Sensors (Basel).

[38] Picerno P, Iosa M, D'Souza C, Benedetti MG, Paolucci S, Morone G (2021). Wearable inertial sensors for human movement analysis: a five-year update. Expert Rev Med Devices.

[39] Iosa M, Picerno P, Paolucci S, Morone G (2016). Wearable inertial sensors for human movement analysis. Expert Rev Med Devices.

[40] Yen CT, Liao JX, Huang YK (2020). Human daily activity recognition performed using wearable inertial sensors combined with deep learning algorithms. IEEE Access.

[41] Huynh-The T, Hua CH, Kim DS (2019). Visualizing inertial data for wearable sensor based daily life activity recognition using convolutional neural network. Annu Int Conf IEEE Eng Med Biol Soc.

[42] Mustafa Z (2023). A study of machine learning techniques based on human daily living activities via inertial sensors.

[43] Ronald M, Poulose A, Han DS (2021). iSPLInception: an Inception-ResNet deep learning architecture for human activity recognition. IEEE Access.

[44] Poulose A, Kim JH, Han DS (2022). HIT HAR: human image threshing machine for human activity recognition using deep learning models. Comput Intell Neurosci.

[45] García-Luna MA, Jimenez-Olmedo JM, Pueo B, Manchado C, Cortell-Tormo JM (2024). Concurrent validity of the ergotex device for measuring low back posture. Bioengineering (Basel).

[46] Jimenez-Olmedo JM, Tortosa-Martínez J, Cortell-Tormo JM, Pueo B (2024). Assessing the validity of the ergotex IMU in joint angle measurement: a comparative study with optical tracking systems. Sensors (Basel).

[47] Kiranyaz S, Avci O, Abdeljaber O, Ince T, Gabbouj M, Inman DJ (2021). 1D convolutional neural networks and applications: a survey. Mech Syst Signal Process.

[48] Heaton J (2018). Ian goodfellow, yoshua bengio, and aaron courville: deep learning. Genet Program Evolvable Mach.

[49] Hochreiter S, Schmidhuber J (1997). Long short-term memory. Neural Comput.

[50] Park S, Kwak N (2017). Analysis on the dropout effect in convolutional neural networks. Lecture Notes in Computer Science (including subseries Lecture Notes in Artificial Intelligence and Lecture Notes in Bioinformatics).

[51] Srivastava N, Hinton G, Krizhevsky A, Sutskever I, Salakhutdinov R (2014). Dropout: a simple way to prevent neural networks from overfitting. JMLR.

[52] Salehin I, Kang DK (2023). A review on dropout regularization approaches for deep neural networks within the scholarly domain. Electronics (Switzerland).

[53] Jin J, Dundar A, Culurciello E (2015). Flattened convolutional neural networks for feedforward acceleration.

[54] Christlein V, Spranger L, Seuret M, Nicolaou A, Kral P, Maier A (2019). Deep generalized max pooling.

[55] Lee CY, Gallagher P, Tu Z (2018). Generalizing pooling functions in CNNs: mixed, gated, and tree. IEEE Trans Pattern Anal Mach Intell.

[56] Banerjee C, Mukherjee T, Pasiliao E (2019). An empirical study on generalizations of the RelU activation function.

[57] Zhu D, Lu S, Wang M, Lin J, Wang Z (2020). Efficient precision-adjustable architecture for softmax function in deep learning. IEEE Trans Circuits Syst II Express Briefs.

